# Another Brick in the Wall: a Rhamnan Polysaccharide Trapped inside Peptidoglycan of *Lactococcus lactis*

**DOI:** 10.1128/mBio.01303-17

**Published:** 2017-09-12

**Authors:** Irina Sadovskaya, Evgeny Vinogradov, Pascal Courtin, Julija Armalyte, Mickael Meyrand, Efstathios Giaouris, Simon Palussière, Sylviane Furlan, Christine Péchoux, Stuart Ainsworth, Jennifer Mahony, Douwe van Sinderen, Saulius Kulakauskas, Yann Guérardel, Marie-Pierre Chapot-Chartier

**Affiliations:** a Équipe BPA, Université du Littoral Côte d’Opale, Institut Régional Charles Violette EA 7394, USC Anses-ULCO, Boulogne-sur-Mer, France; b Institute for Biological Sciences, National Research Council of Canada, Ottawa, Ontario, Canada; c Micalis Institute, INRA, AgroParisTech, Université Paris-Saclay, Jouy-en-Josas, France; d INRA, UMR 1313 GABI, Plate-forme MIMA2, Jouy-en-Josas, France; e School of Microbiology, University College Cork, Cork, Ireland; f APC Microbiome Institute, University College Cork, Cork, Ireland; g Université de Lille, CNRS, UMR 8576, UGSF-Unité de Glycobiologie Structurale & Fonctionnelle, Lille, France; University of Queensland; KUMC

**Keywords:** HR-MAS NMR, *Lactococcus*, cell wall, polysaccharides, rhamnan

## Abstract

Polysaccharides are ubiquitous components of the Gram-positive bacterial cell wall. In *Lactococcus lactis*, a polysaccharide pellicle (PSP) forms a layer at the cell surface. The PSP structure varies among lactococcal strains; in *L. lactis* MG1363, the PSP is composed of repeating hexasaccharide phosphate units. Here, we report the presence of an additional neutral polysaccharide in *L. lactis* MG1363 that is a rhamnan composed of α-l-Rha trisaccharide repeating units. This rhamnan is still present in mutants devoid of the PSP, indicating that its synthesis can occur independently of PSP synthesis. High-resolution magic-angle spinning nuclear magnetic resonance (HR-MAS NMR) analysis of whole bacterial cells identified a PSP at the surface of wild-type cells. In contrast, rhamnan was detected only at the surface of PSP-negative mutant cells, indicating that rhamnan is located underneath the surface-exposed PSP and is trapped inside peptidoglycan. The genetic determinants of rhamnan biosynthesis appear to be within the same genetic locus that encodes the PSP biosynthetic machinery, except the gene *tagO* encoding the initiating glycosyltransferase. We present a model of rhamnan biosynthesis based on an ABC transporter-dependent pathway. Conditional mutants producing reduced amounts of rhamnan exhibit strong morphological defects and impaired division, indicating that rhamnan is essential for normal growth and division. Finally, a mutation leading to reduced expression of *lcpA*, encoding a protein of the LytR-CpsA-Psr (LCP) family, was shown to severely affect cell wall structure. In *lcpA* mutant cells, in contrast to wild-type cells, rhamnan was detected by HR-MAS NMR, suggesting that LcpA participates in the attachment of rhamnan to peptidoglycan.

## IMPORTANCE

In the cell wall of Gram-positive bacteria, the peptidoglycan sacculus is considered the major structural component, maintaining cell shape and integrity. It is decorated with other glycopolymers, including polysaccharides, the roles of which are not fully elucidated. In the ovococcus *Lactococcus lactis*, a polysaccharide with a different structure between strains forms a layer at the bacterial surface and acts as the receptor for various bacteriophages that typically exhibit a narrow host range. The present report describes the identification of a novel polysaccharide in the *L. lactis* cell wall, a rhamnan that is trapped inside the peptidoglycan and covalently bound to it. We propose a model of rhamnan synthesis based on an ABC transporter-dependent pathway. Rhamnan appears as a conserved component of the lactococcal cell wall playing an essential role in growth and division, thus highlighting the importance of polysaccharides in the cell wall integrity of Gram-positive ovococci.

## INTRODUCTION

The cell wall of Gram-positive bacteria consists of a thick peptidoglycan sacculus decorated with wall teichoic acids (WTA), polysaccharides, and proteins (1). Cell wall polysaccharides (CWPS), when exposed at the outer surface of bacterial cells, mediate crucial bacterial interactions with the environment such as adhesion to abiotic surfaces, specific interactions with other microorganisms or with eukaryotic host cells, and adsorption of infecting bacteriophages (1). Additionally, these so-called “secondary cell wall glycopolymers” may play essential roles in morphogenesis and cell division (2, 3).

The biosynthetic pathway of polysaccharide is generally encoded by large gene clusters encoding glycosyltranferases, proteins involved in chain polymerization and proteins involved in polysaccharide chain or subunit transport across the cytoplasmic membrane (4). Three distinct mechanisms of polymerization/export have been described in both Gram-negative and Gram-positive bacteria. These include Wzx/Wzy-, ATP-binding cassette (ABC) transporter-, and synthase-dependent pathways (4–6). In the two former pathways, polysaccharide (subunit) synthesis is initiated on a lipid acceptor, while such an acceptor is not involved in the latter. In the Wzx/Wzy-dependent pathway, the polysaccharide subunit is synthesized in the cytoplasm on a cell membrane-associated undecaprenyl-phosphate acceptor moiety, transferred by the flippase Wzx outside the cytoplasmic membrane, and finally assembled into the mature polysaccharide by the polymerase Wzy. In the ABC transporter-dependent pathway, the entire polysaccharide chain is assembled on the lipid carrier before being transported across the inner membrane by an ABC transporter. For synthase-dependent secretion, the polysaccharide is polymerized and exported across the inner membrane by the synthase protein. In addition, in Gram-positive bacteria, members of the LytR-Cps2A-Psr protein family have been identified as the transferases involved in the anchoring of secondary cell wall glycopolymers, including WTA and CWPS (or capsular polysaccharide [CPS]) onto peptidoglycan (7–10).

*Lactococcus lactis* is a Gram-positive lactic acid bacterium widely used in dairy fermentations. We have previously reported that *L. lactis* MG1363 possesses a CWPS that constitutes a bacterial surface layer that was named the polysaccharide pellicle (PSP) (11, 12). This PSP is composed of branched hexasaccharide subunits that contain Glc, Gal, GlcNAc, and Rha and are linked by phosphodiester bonds. In contrast to previously characterized lactococcal exopolysaccharides that are released into the surroundings and confer textural properties on the strains that produce them, PSP is most likely covalently linked to the cell wall. The PSP not only provides a protective barrier against phagocytosis by murine macrophages but also acts as the receptor for members of various lactococcal phage groups, allowing their adsorption through specific recognition events (13–15).

**FIG 1:**
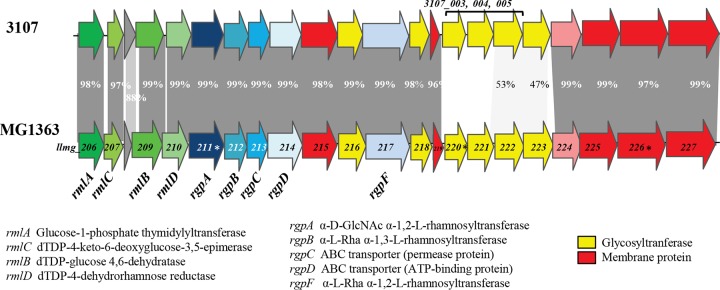
Alignment of the chromosomal gene clusters encoding the biosynthetic machinery for CWPS production in *L. lactis* MG1363 and 3107. Mutated genes are marked with asterisks.

The genes encoding PSP biosynthesis are located in a large chromosomal gene cluster in *L. lactis* (11) that is composed of a highly conserved region, as well as a more variable region (Fig. 1). On the basis of sequence similarity analysis of the variable region among the currently available *L. lactis* genome sequences, three major CWPS types (A, B, and C) were defined (16). Interestingly, a correlation exists between the CWPS genotype and the host range of the 936-type phages tested. Furthermore, within the C-type genotype, five different subtypes (designated C1 to C5) have been discerned on the basis of sequence conservation within the more variable region of the *cwps* locus (14). In addition to the PSP structure of *L. lactis* MG1363, those of two other strains (SMQ-388 and 3107, both of the C type) have been determined, thereby confirming that genetic variation of the *cwps* locus among *L. lactis* strains indeed corresponds to structural diversity of their respective PSPs (13, 14). The PSP structures of the three aforementioned strains are sugar phosphate polysaccharides, but the three differ by their oligosaccharide repeating units. Notably, it was shown that the variations in PSP structure are major determining factors in bacteriophage sensitivity (14, 15). High-resolution magic-angle spinning nuclear magnetic resonance (HR-MAS NMR) experiments performed with intact whole bacterial cells allowed us to reveal surface-exposed CWPS and confirmed the surface localization of PSP (14).

We observed that, apart from the PSP, crude extracts of *L. lactis* strains contained variable amounts of an additional, rhamnose-rich polysaccharide component. Furthermore, in a PSP-negative mutant, HR-MAS NMR experiments detected signals corresponding to another carbohydrate molecule (14). Taken together, these observations prompted us to investigate this apparently novel CWPS. In the present study, we show that *L. lactis* MG1363 contains an additional neutral polysaccharide strongly associated with the cell wall and composed of α-l-Rha trisaccharide repeating units. Rhamnan with an identical structure was also purified from *L. lactis* strain 3107. We show here that, in contrast to the PSP, rhamnan is not exposed at the bacterial surface and is likely embedded within the peptidoglycan. On the basis of gene sequence analysis and experimental results obtained with constructed mutants, we propose a model of the synthesis and cell wall attachment of rhamnan. From the analysis of *L. lactis* mutants producing reduced amounts of rhamnan, we conclude that rhamnan synthesis is essential for normal growth and division.

## RESULTS

### Purification and structural elucidation of a rhamnan from *L. lactis* MG1363 and 3107.

In our previous study (11), cell wall-associated carbohydrates were extracted from the defatted cell walls of *L. lactis* MG1363 by trichloroacetic acid (TCA) treatment. The TCA extract contained the PSP as a major component. Subsequent treatment of the cell debris with hot diluted HCl, followed by hydrogen fluoride (HF) treatment and purification by gel filtration chromatography, revealed the presence of a different neutral polysaccharide with a molecular mass of ∼3 to 5 kDa. This polysaccharide contained Rha as a major component and traces of Glc, Gal, and GlcNAc. The absolute configuration of Rha was determined as l-Rha.

The chemical structure of this novel polysaccharide was established by a combination of methylation analysis, one-dimensional (1D) and 2D NMR techniques, and matrix-assisted laser desorption ionization–time of flight mass spectrometry (MALDI-TOF MS) analysis. Methylation analysis identified the presence of two major components, 2- and 3-substituted Rha, in an approximate molar ratio of 2:1, indicating a linear structure. Among the minor components, a 2,4-disubstituted Rha, terminal Glc, terminal GlcNAc, and substituted HexNAc were also identified. Terminal Rha, however, was absent in methylation analysis of rhamnan preparations.

**FIG 2:**
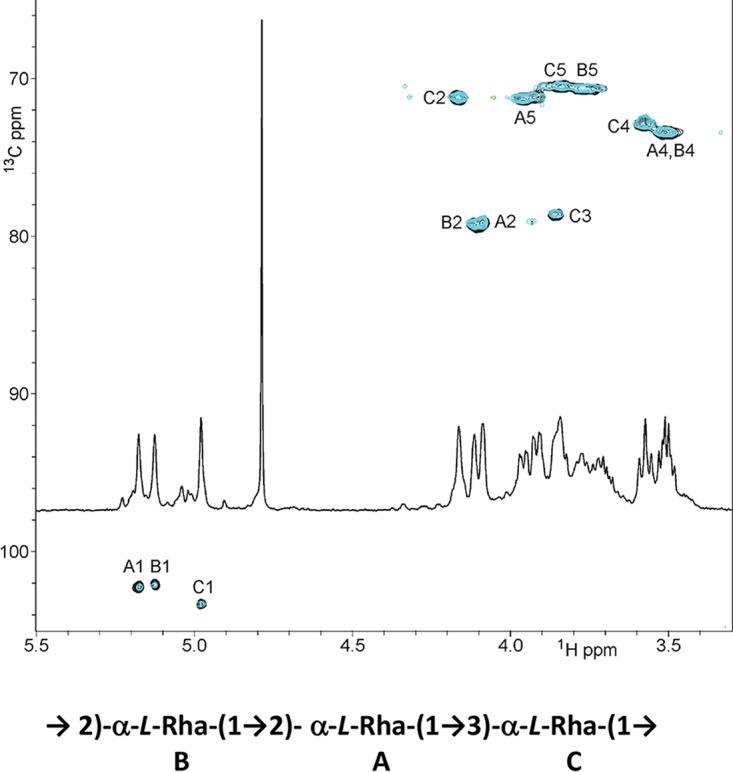
NMR analysis of rhamnan. HMQC spectra of rhamnan purified from *L. lactis* MG1363 (black) and 3107 (blue) and proposed structure of the repeating unit of the major rhamnan from both strains.

The ^1^H and ^1^H-^13^C heteronuclear single-quantum coherence (HSQC) NMR spectra revealed the presence of three anomeric signals A, B, and C (Fig. 2). An *N*-acetyl signal was also observed in the ^1^H NMR spectrum. Complete assignment of 2D NMR spectra (Table 1) and analysis of nuclear Overhauser effect spectroscopy (NOESY) and heteronuclear multiple-quantum coherence (HMBC) data led to the identification of a linear trisaccharide repeating unit containing two 2-linked and one 3-linked α-l-Rha residues corresponding to a rhamnan polysaccharide (Fig. 2).

**TABLE 1:** NMR data^a^ for the rhamnan of *L. lactis* MG1363 (and 3107)

α-Rha unit	Chemical shift (ppm)					
	H/C-1	H/C-2	H/C-3	H/C-4	H/C-5	H/C-6
A						
H	5.17	4.09	3.96	3.51	3.84	1.32
C	102.20	79.00	71.20	73.30	70.40	17.80
B						
H	5.12	4.11	3.92	3.50	3.72	1.29
C	102.10	79.20	71.10	73.30	70.50	17.80
C						
H	4.98	4.16	3.85	3.57	3.77	1.28
C		71.10	78.40	72.80	70.50	17.80

a Spectra were recorded in D_2_O at 25°C with a 500-MHz spectrometer.

**FIG 3:**
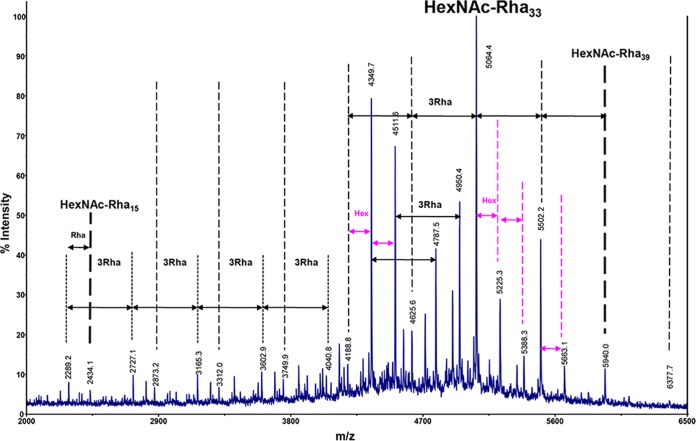
MALDI-TOF MS spectrum of rhamnan extracted from *L. lactis* MG1363. All *m/z* values correspond to [M + Na]^+^ adducts.

MALDI-TOF MS analysis of the rhamnan preparation showed a complex pattern of signals ranging from *m/z* 2,289.2 to *m/z* 6,377.7 (Fig. 3). This is in agreement with the elution profile determined by gel filtration (molecular mass, 3 to 5 kDa). A major family of signals ranging from *m/z* 2,434.1 to *m/z* 5,940.0 with 438-m.u. (mass unit) increments was tentatively assigned to [M + Na]^+^ ion adducts of HexNAc-(dHex)_15–39_ with a maximum abundance for a compound identified as an 11-trisaccharidic repeating unit (HexNAc_1_dHex_33_ at *m/z* 5,064.4, theoretical *m/z* 5,063.9904). On the basis of monosaccharide and NMR analyses, HexNAc and dHex residues were assigned as GlcNAc and Rha, respectively. A population of molecules containing Hex was also present in the MALDI-TOF MS spectrum. Since a terminal rhamnose was not identified upon methylation analysis, we hypothesize that GlcNAc or Glc residues could be present at the nonreducing ends of the rhamnan polysaccharide chains.

We also examined the CWPS content of another strain, *L. lactis* 3107, that possesses a C-type CWPS biosynthesis cluster like MG1363 but belongs to the C2 subtype, whereas MG1363 belongs to the C1 subtype (Fig. 1). *L. lactis* 3107 was previously shown to possess a PSP that is also a phosphopolysaccharide yet with repeating pentasaccharide units instead of the hexasaccharide found in *L. lactis* MG1363 (14). Employing a protocol similar to the one used for MG1363, a rhamnan identical to that of MG1363 (as determined by NMR) was extracted from *L. lactis* 3107 (Fig. 2).

### Synthesis of rhamnan in mutants devoid of PSP.

**FIG 4:**
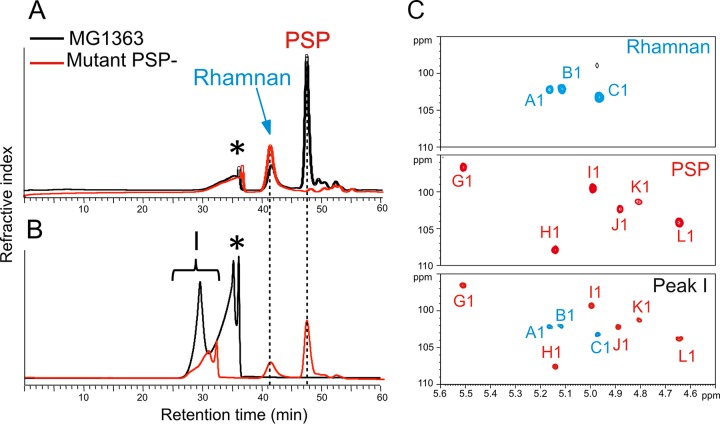
SEC-HPLC and NMR analyses of CWPS extracted from wild-type and PSP-negative mutant (VES5748) *L. lactis* MG1363. (A) SEC-HPLC separation of CWPS extracted by HF from MG1363 showing two polysaccharide signals identified as rhamnan and PSP-derived oligosaccharides, whereas VES5348 shows only rhamnan. (B) SEC-HPLC separation of cell wall products from MG1363 digested with mutanolysin showing a major polysaccharide signal (peak I in black). When hydrolyzed with HF, purified peak I regenerated rhamnan and PSP-derived oligosaccharides (red line). (C) Comparison of the anomeric regions of the ^1^H-^13^C HSQC NMR spectra of different compounds confirming that peak I contained both rhamnan and PSP-derived oligosaccharides. The attribution of PSP monosaccharide signals was based on previous NMR identification of intact and HF-generated oligosaccharides from the PSP (11, 14). Asterisks indicate nonpolysaccharide compound.

A mutant derived from *L. lactis* MG1363 and devoid of a PSP (VES5748) had previously been isolated (11). VES5748 was shown to carry a point mutation in *llmg_0226*, which is located at the distal end of the polysaccharide gene cluster and encodes a putative transmembrane protein of unknown function (Fig. 1). This mutant was shown by biochemical analysis, as well as microscopy observations, to lack a PSP (11, 12), and we thus sought to investigate whether or not VES5748 synthesizes rhamnan. To achieve this, polysaccharides were extracted from cell walls by HF treatment and further separated by size exclusion–high-performance liquid chromatography (SEC-HPLC) (Fig. 4A). For wild-type *L. lactis* MG1363, composition analysis and MALDI-TOF MS analysis of the two major peaks revealed that they contained rhamnan and oligosaccharides derived from the PSP by cleavage of phosphodiester bonds by HF treatment, respectively (data not shown). When cell walls from PSP-negative mutant VES5748 were treated with HF, no PSP hexasaccharides were detected, as expected, whereas rhamnan was still present (Fig. 4A). Rhamnan extracted from the mutant strain was identical to that from the wild-type strain, with a similar length distribution and HexNAc and Hex substituents, as shown by MALDI-TOF MS analysis (data not shown). A similar SEC-HPLC profile was obtained with another PSP-negative mutant derived from *L. lactis* NZ9000 (an MG1363 derivative; see Table S1 in the supplemental material) obtained by inactivation of the glycosyltransferase gene *LLNZ_01145* (14) (corresponding to *llmg_0220* in Fig. 1) (uninduced *L. lactis* NZ9000-GT1/pPTPiC2 strain in Table S1) (data not shown). These results indicate that rhamnan can be synthesized independently of PSP biosynthesis.

10.1128/mBio.01303-17.1 TABLE S1  *L. lactis* strains and plasmids used in this study. Download TABLE S1, DOCX file, 0.02 MB. Copyright © 2017 Sadovskaya et al. 2017 Sadovskaya et al. This content is distributed under the terms of the Creative Commons Attribution 4.0 International license.

### Localization of rhamnan in the cell wall of *L. lactis* MG1363.

**FIG 5:**
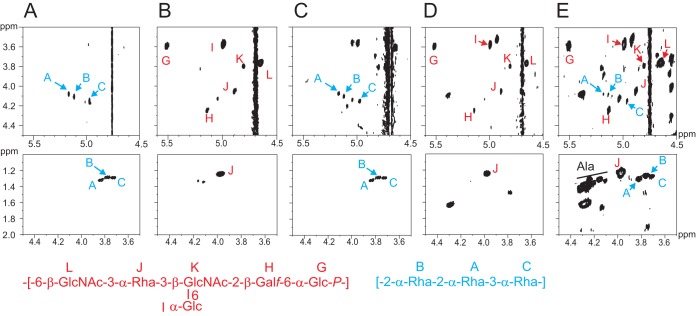
NMR analysis of whole wild-type and derivative mutant *L. lactis* NZ9000 bacteria. (A) Details of ^1^H-^1^H COSY spectra obtained by liquid NMR of rhamnan purified from *L. lactis* NZ9000. (B) HR-MAS NMR of wild-type NZ9000 cells. (C) HR-MAS NMR of NZ9000 PSP-negative mutant (noninduced NZ9000-GT1/pPTPiC2) cells. (D) HR-MAS NMR of NZ9000 *lcpB* deletion mutant (PAR152) cells. (E) HR-MAS NMR of NZ9000 conditional *lcpA* mutant (VES6320) cells. H1-H2 ^3^*J*_H-H_ (top panels) and H5-H6 ^3^*J*_H-H_ (bottom panels) correlation signals from PSP and rhamnan are shown at the top. The structures of the repeating units of the PSP (red) and rhamnan (blue) from MG1363/NZ9000 are shown at the bottom.

HR-MAS NMR represents a direct analysis of exposed components at the surface of intact bacterial cells. This technique previously allowed us to identify PSP at the surface of wild-type *L. lactis* NZ9000, as shown by the HR-MAS ^1^H-^1^H correlation spectroscopy (COSY) spectra (Fig. 5B). Furthermore, the HR-MAS ^1^H-^13^C HSQC NMR spectrum of *L. lactis* PSP-negative NZ9000 cells (noninduced *L. lactis* NZ9000-GT1/pPTPiC2) established that the inactivation of the glycosyltransferase-encoding gene *LLNZ_01145* caused the disappearance of the PSP at the cell surface (14). However, the observation of unidentified NMR carbohydrate signals suggested the presence of another CWPS that was not identified at that time. Here, by further analyzing the cell surface by ^1^H-^1^H COSY experiments and comparing the data with those obtained from the purified PSP (11) and from the newly identified rhamnan, we could not only confirm the disappearance of the PSP but also identify rhamnan at the surface of *L. lactis* NZ9000 PSP-negative mutant cells (Fig. 5). Indeed, as shown in Fig. 5C, the ^1^H-^1^H COSY spectrum obtained permitted us to identify the H1-H2 and H5-H6 ^3^*J*_H-H_ correlation signals from three monosaccharides, A, B, and C, that show chemical shift values identical to those obtained from the purified rhamnan by liquid NMR experiments (Fig. 5A). These results indicate that, in the absence of a PSP, rhamnan either becomes exposed at the bacterial surface or becomes more flexible inside the cell wall. Thus, rhamnan appears to be located underneath the PSP and embedded and/or trapped inside the peptidoglycan layer of *L. lactis*.

### Rhamnan biosynthesis is encoded in the same chromosomal gene cluster as the PSP.

**FIG 6:**
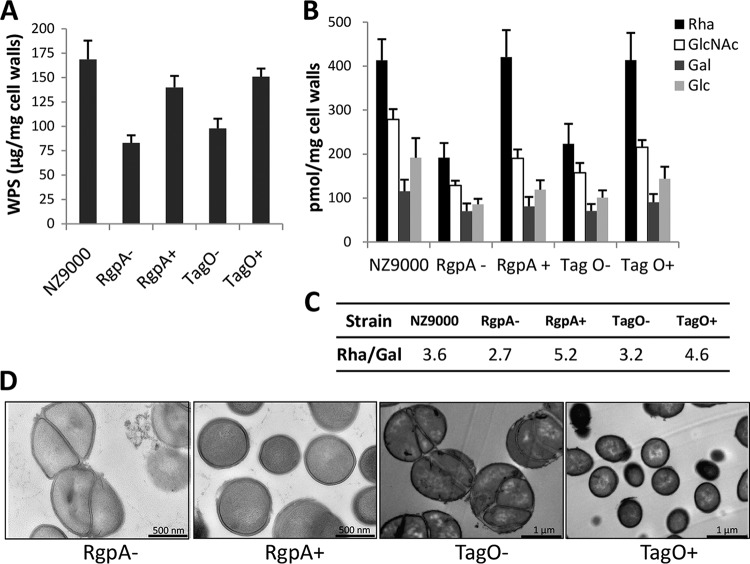
*rgpA* and *tagO* are involved in rhamnan synthesis in *L. lactis* MG1363. (A to C) Quantitative levels and composition of total CWPS (containing rhamnan and the PSP) in wild-type *L. lactis* NZ9000 and *rgpA* and *tagO* conditional mutants. (A) Total amount of CWPS. (B) Amount of each monosaccharide. (C) Rha/Gal molar ratio. *rgpA* and *tagO* conditional mutants were grown without nisin (−) or with nisin at 0.1 ng/ml (+). CWPS were extracted from cell walls with HF for 48 h at 4°C. Monosaccharides were quantified by HPAEC-PAD after TFA hydrolysis. Mean values were calculated from two independent cultures, each analyzed three times. (D) TEM of *L. lactis rgpA* and *tagO* conditional mutants grown without (−) or with (+) nisin.

Comparison of the gene cluster encoding CWPS synthesis in *L. lactis* strains MG1363 and 3107 revealed a conserved 5′-located region and a more variable 3′-located region (Fig. 1). In addition, as reported in our previous study, genetic swapping of a part of the PSP clusters between NZ9000 and 3107 had demonstrated that the variable part is involved in PSP synthesis (14). Therefore, we hypothesized that the rhamnan biosynthetic machinery is encoded by the part conserved between the two strains, i.e., the genes located in the 5′-proximal region of the cluster. In this region, three genes encoding putative rhamnosyltransferases (*rgpA*, *rgpB*, and *rgpF*) are present. They are located downstream of four genes, named *rmlA* to *rmlD*, that represent clear homologs of genes required for rhamnose precursor (dTDP-Rha) synthesis (17). Despite several attempts, we did not succeed in inactivating any of the *rgpA*, *rgpB*, and *rgpF* genes. A conditional mutant in which the *rgpA* gene was placed under the control of the nisin-inducible promoter was generated in NZ9000, a derivative of MG1363 that carries the *nisRK* genes required for nisin-mediated gene induction (18). Total CWPS were extracted by HF from cell walls of the *rgpA* conditional mutant grown with or without nisin, and their composition and amount were determined following acid hydrolysis by high-performance anion-exchange chromatography coupled with pulsed amperometric detection (HPAEC-PAD). We observed a 50% decrease in the total amount of CWPS in the cell wall of the *rgpA* mutant grown without nisin compared to that in the wild-type strain (Fig. 6A). The incomplete switching off of CWPS synthesis in the absence of nisin is probably due to the leakiness of the nisin-inducible promoter (19). Compositional analysis showed a decrease in all of the monosaccharides constituting rhamnan (mainly Rha), as well as the PSP (composed of Glc, GlcNAc, Rha, and Gal in a ratio of 2:2:1:1) (Fig. 6B). In the presence of nisin, the total amount of CWPS reached the level of the wild-type strain. These results indicate that when the *rgpA* expression level is reduced, the synthesis of both rhamnan and the PSP is affected. However, the molar ratio of Rha/Gal was lower in the *rgpA* mutant without nisin than in the wild type and higher in the *rgpA* mutant with nisin, indicating that rhamnan synthesis is affected more by the *rgpA* transcription level than PSP synthesis is (Fig. 6C). These results confirm that *rgpA*, localized in the same gene cluster as the genes involved in PSP synthesis, is involved in rhamnan synthesis.

In the absence of nisin, the *rgpA* conditional mutant exhibited poor growth. Transmission electron microscopy (TEM) revealed strongly altered cell morphology with defects in cell septation and division (Fig. 6D). In the presence of nisin, normal cell morphology and division were restored in this strain (Fig. 6D). These observations indicate that rhamnan is an essential component of the *L. lactis* cell wall.

### Identification of the initial transferase involved in rhamnan synthesis.

In the Wzx/Wzy- and ABC transporter-dependent pathways, the first step of polysaccharide biosynthesis in bacteria consists of the transfer of a phosphosugar to an undecaprenylphosphate lipid carrier in the bacterial membrane, catalyzed by an initial transferase or primase. A sequence identity search in the MG1363 gene cluster encoding CWPS biosynthesis (shown in Fig. 1) failed to identify a gene for an appropriate initiating glycosyltransferase. However, a gene was identified (*llmg_1976/llnz_10205*) that encodes a protein with sequence similarity, including a conserved overall topology of 11 transmembrane segments, to proteins of the WecA family, including *Escherichia coli* WecA, which initiates lipopolysaccharide O-antigen synthesis (20), and *Streptococcus mutans* RgpG (21) or *Streptococcus agalactiae* GbsO (2), which both initiate rhamnose-rich polysaccharide synthesis. These enzymes catalyze the transfer of GlcNAc-1-P from UDP-GlcNAc to the undecaprenylphosphate carrier molecule in the bacterial cytoplasm. Thus, the product of *llmg_1976/llnz_010205* (named *tagO*) may act as the initial transferase of rhamnan synthesis. Despite several attempts by single- and double-crossover approaches, inactivation of *llmg_1976* in *L. lactis* MG1363 was unsuccessful. Therefore, a *llmg_1976* conditional mutant (named TIL1116) was constructed by cloning the *tagO* gene under the control of the nisin-inducible promoter and integrating the fusion into the chromosome of strain NZ9000. CWPS were extracted by HF from cell walls of the *tagO* conditional mutant grown with or without nisin. Analysis of the monosaccharide content showed that total CWPS extracted from cell walls was decreased by 40% in the *tagO* conditional mutant grown without nisin compared to the wild type (Fig. 6A), indicating that *tagO* is required for CWPS synthesis. Compositional analysis showed a decrease in all of the monosaccharides constituting rhamnan (mainly Rha), as well as the PSP (Glc, GlcNAc, Rha, and Gal in a ratio of 2:2:1:1) (Fig. 6B). In the presence of nisin, the total amount of CWPS reached the level of the wild-type strain. These results indicate that when the *tagO* expression level is reduced, the synthesis of both rhamnan and PSP is affected, thus confirming that the involvement of *llmg_1976* is in CWPS synthesis and particularly in rhamnan synthesis.

TEM of mutant TIL1116 grown without nisin revealed severe defects in cell septum formation, dramatic alterations of the cell shape, and the formation of large cell clusters (Fig. 6D), further indicating the essential role of CWPS in cell wall biogenesis.

### Attachment of rhamnan to peptidoglycan.

The fact that harsh acid treatments are required to extract CWPS from the *L. lactis* cell wall suggests that these glycopolymers are covalently bound to peptidoglycan. To investigate this issue, we digested cell walls containing peptidoglycan and CWPS with the peptidoglycan hydrolase mutanolysin and separated the soluble products by SEC-HPLC. Depolymerization of cell wall by mutanolysin generated a major signal polysaccharide peak (peak I) (Fig. 4B). Colorimetric analysis established that the other peak observed by SEC-HPLC (asterisk in Fig. 4B) contained only a small amount of carbohydrates (data not shown); thus, its nature was not further investigated. NMR analysis of peak I established that it contained both a PSP and rhamnan, owing to the clear observation of all of the individual monosaccharides of the two polysaccharides (Fig. 4C). Furthermore, multiple signals associated with GlcNAc and MurNAc from peptidoglycan were identified in peak I (data not shown), which strongly suggests that both the PSP and rhamnan are covalently associated with peptidoglycan. When further treated with HF, peak I generated two peaks of lower molecular weight whose elution times by SEC-HPLC corresponded to those of rhamnan and PSP oligosaccharides (Fig. 4B). Altogether, these analyses strongly suggest that PSP and rhamnan polysaccharides are covalently linked together into a single heteropolysaccharide that itself is covalently attached to peptidoglycan. However, we could not establish the exact linkage between the PSP and rhamnan.

LytR-CpsA-Psr (LCP) enzymes have been proposed to catalyze the covalent attachment of secondary polymers such as WTA, as well as CPS, to peptidoglycan in bacilli and staphylococci (7–9), and their enzymatic activity was recently shown by *in vitro* reconstitution (10). In *L. lactis* NZ9000, three *lcp* paralogs, *lcpA* (*llnz_02385*), *lcpB* (*llnz_03815*), and *lcpC* (*llnz_08240*), are present. It is noteworthy that *lcpC* contains a stop codon at the beginning of the open reading frame that renders it nonfunctional. The other two genes, *lcpA* and *lcpB*, are located outside the *cwps* gene cluster and have a monocistronic organization. The *lcpB* gene was successfully deleted from NZ9000, in contrast to *lcpA*, suggesting an essential role for LcpA in the growth and/or survival of *L. lactis* NZ9000. A conditional mutant of NZ9000 was obtained by cloning *lcpA* under the control of the nisin-inducible promoter P_*nisA*_. Also, a conditional mutant was obtained in the NZ9000Δ*lcpB* background.

Intact cells of the *lcp* mutants were examined by HR-MAS NMR. Identical spectra were obtained for the Δ*lcpB* mutant and wild-type NZ9000, indicating that the Δ*lcpB* mutant displays the PSP pellicle on its surface like NZ9000 (Fig. 5B and D). When the NZ9000 *lcpA* conditional mutant grown in the absence of nisin was examined, the NMR spectrum obtained contained PSP signals but also signals corresponding to rhamnan (Fig. 5E). An identical spectrum was obtained for the *lcpA lcpB* double mutant (data not shown). These observations indicate the presence of a more flexible rhamnan and thus possibly of a (partially) free rhamnan in the cell wall of the *lcpA* mutant that could result from deficient attachment of rhamnan to peptidoglycan because of the reduced activity of LcpA. In conclusion, all of these data strongly suggest that LcpA has a major role compared to LcpB in attaching the rhamnan to the peptidoglycan. Furthermore, the observation of numerous other signals, including methyl groups of Ala residues (Fig. 5E), suggests that the reduced expression of LcpA causes further disorganization in the cell wall.

## DISCUSSION

In this study, we found that the cell wall of *L. lactis* MG1363 contains two polysaccharides, an acidic phosphopolysaccharide that forms a thin compact outer layer named the PSP (11) and a neutral polysaccharide, described in this study, that is present in the inner layers of the cell wall. The latter neutral CWPS was shown to be a linear α-l-rhamnan composed of a trisaccharide repeating unit (2-α-Rha--2-α-Rha--3-α-Rha). A rhamnan with an identical structure was also purified from another strain, *L. lactis* 3107, that is endowed with a PSP with a different structure. This is the first report of the presence of a rhamnan component in *L. lactis* cell walls.

Rhamnose-rich polysaccharides have been previously recognized as important cell wall components of streptococci. Their structural diversity between bacterial species was initially used for the Lancefield serological classification and identification of streptococci. In addition to their immunological properties, they have a variety of functions in phage adsorption, coaggregation between bacterial species in biofilms, and host colonization (22). In several *Streptococcus* species, including *Streptococcus pyogenes* (group A) (23), *S. mutans* (24), *S. dysgalactiae* (25), and *S. uberis* (26), these polysaccharides consist of linear polymers of α-1,2- and α-1,3-l-Rha with a monosaccharidic side chain consisting of Glc or GlcNAc. In *S. agalactiae* (group B), it is a multiantennary branched structure (27). *L. lactis* MG1363 has a more complex CWPS content with two polymeric chains with different chemical structures, both containing Rha.

*L. lactis* strains possess a large chromosomal gene cluster encoding CWPS biosynthesis, comprising a highly conserved and a variable region. On the basis of sequence similarity or differences in the variable region, *L. lactis* strains were classified into three major groups (types A, B, and C) (16), with five subtypes (C1 to C5) distinguished in the C-type genotype (14). We have previously shown that the two *L. lactis* strains, 3107 and MG1363, belonging to two different C subtypes synthesize a PSP with a different chemical structure (14). Additionally, by swapping the PSPs of the two strains, it was concluded that the variable part of the *cwps* gene cluster is involved in PSP synthesis. In the present study, we found a rhamnan with an identical structure in strains MG1363 and 3107. The results obtained by *rgpA* inactivation show that the *cwps* gene cluster is also involved in rhamnan synthesis. Therefore, we propose that the conserved part of the *cwps* gene cluster, i.e., the genes located in the 5′ region, is involved in rhamnan biosynthesis. The genes annotated as *rmlA* to *rgpF* (Fig. 1) are highly conserved in a number of *L. lactis* strains belonging to the CWPS C- and B-type groups (16), suggesting that rhamnan is a conserved component of the *L. lactis* cell wall, at least in the strains belonging to either of these two groups.

**FIG 7:**
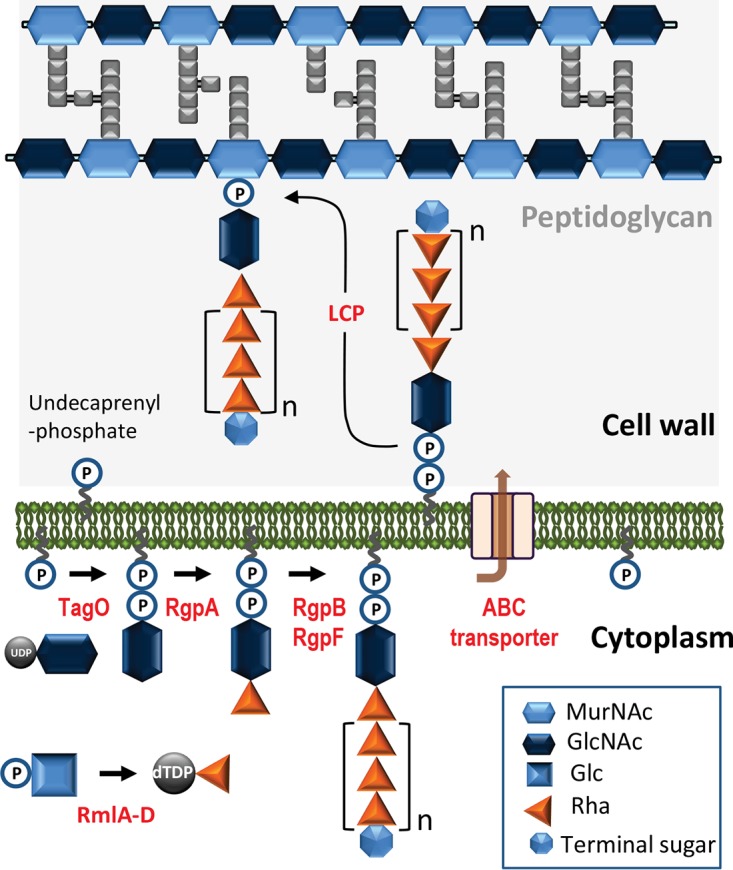
Model of rhamnan biosynthesis in *L. lactis*. The rhamnan chain is assembled on a lipid carrier (undecaprenylphosphate) in the bacterial cytoplasm. Proteins RmlA to RmlD synthesize dTDP-l-Rha precursor. TagO is the initial transferase catalyzing the transfer of GlcNAc onto undecaprenylphosphate. RgpA adds a first l-Rha, and then RgpB and RgpF are involved in the processive elongation of the chain, catalyzing the transfer of l-Rha into positions 3 and 2, respectively, of the previous l-Rha. A putative terminal sugar (GlcNAc or Glc, added by an unknown enzyme) terminates the synthesis of the chain. An ABC transporter exports the rhamnan from the cytoplasm to the extracellular side of the cytoplasmic membrane. Finally, phosphorhamnan chains are transferred onto peptidoglycan chains by LCP protein(s), with LcpA playing the main role.

On the basis of our experimental results and sequence homology analysis, we propose the following model of rhamnan biosynthesis, involving an ABC transporter-dependent pathway (Fig. 7). Synthesis commences with the transfer of GlcNAc on undecaprenylphosphate catalyzed by the TagO ortholog, a UDP-*N*-acetylglucosamine:undecaprenyl-P *N*-acetylglucosaminyl 1-P transferase, encoded outside the CWPS biosynthesis gene cluster. The *rmlABCD* genes encode the four enzymes known to be involved in rhamnose precursor dTDP-l-Rha synthesis (17). Subsequently, the rhamnosyltransferase RgpA transfers the first rhamnosyl residue on the GlcNAc-primed lipid intermediate acceptor and initiates the synthesis of the rhamnan chain. Two other rhamnosyltransferases encoded by *rgpB* and *rgpF* elongate the chain, catalyzing the transfer of l-Rha into positions 3 and 2, respectively, of the previous l-Rha, until an average length of 33 Rha residues (corresponding to 11 repeating units) is reached. Polymerization is terminated by the addition of a GlcNAc or Glc residue at the nonreducing end of the chain, catalyzed by an unknown enzyme. The genes *rgpC* and *rgpD* encode two proteins, a permease and an ATP-binding protein, respectively, constituting an ABC transport system responsible for the export of the elongated rhamnan chain outside the cytoplasmic membrane. Following export, the rhamnan is most probably anchored covalently to peptidoglycan acceptor sites via phosphodiester linkage to MurNAc or GlcNAc residues of the glycan chains, with LcpA being the main ligase involved.

From methylation analysis and MALDI-TOF MS data, we deduced that the nonreducing end of the rhamnan could be terminated with single Glc or GlcNAc residues. Previous studies of polymannose O antigens from *E. coli* have shown that modifications of the nonreducing end of the polysaccharide chains determine chain length and couple chain termination to polymer export via an ABC transporter (28). Nonreducing terminal modifications of different types have also been found in other glycans in Gram-positive bacteria such as the CWPS of *Lactobacillus casei* BL23 (29) or the glycosidic moiety of S layers in a number of species of Gram-positive members of the family *Bacillaceae* (6). Thus, more generally, nonreducing terminal modifications are proposed to be involved in the termination of chain polymerization and possibly in the coordination of export through the inner membrane via ABC transporters (6). In the model that we propose for rhamnan synthesis with an ABC transporter-dependent pathway (Fig. 7), we suggest that a terminal GlcNAc or Glc at the nonreducing end of the *L. lactis* rhamnan performs this function.

Our results obtained by HR-MAS NMR analysis of whole bacterial cells, in combination with our previous observations by TEM and atomic force microscopy, provide information regarding the spatial organization of CWPS in the cell wall. All of these observations show that the PSP is exposed at the bacterial surface and forms an outer layer that covers the bacterial cells. In contrast, according to HR-MAS NMR experiments, rhamnan could be detected only in the absence of a PSP and thus appears to be trapped and embedded in the peptidoglycan network. Both rhamnan and the PSP required strong acid treatment to be extracted from the cell walls, suggesting that they are covalently bound to the cell wall. Our data obtained by NMR analysis of the main polysaccharidic product generated by mutanolysin digestion of purified cell walls strongly suggest that the PSP and rhamnan polysaccharides are covalently linked together into a single heteropolysaccharide that itself is covalently attached to peptidoglycan. Further studies will determine the nature of the linkage between PSP and rhamnan and the enzyme catalyzing this linkage.

Proteins of the LCP family have been proposed to catalyze the transfer of secondary cell wall glycopolymers (including WTA, as well as CPS), after transport/flipping outside the cytoplasmic membrane, from undecaprenyl-phosphate precursor onto amino sugar constituents of peptidoglycan chains (7–9). Recently, the enzymatic activity of LCP proteins from *S. aureus* has been demonstrated *in vitro* in a reconstituted system with chemoenzymatically synthesized WTA and peptidoglycan substrates (10). In *L. lactis* NZ9000/MG1363, three *lcp* paralogs are present but only two appear to be functional. In this study, we found that the *lcpB* gene can be deleted without any associated phenotype, whereas *lcpA* is apparently essential. By HR-MAS NMR examination of conditional mutant cells with lowered expression of *lcpA*, we detected rhamnan concomitantly with a PSP, revealing a surface-exposed and/or a more flexible rhamnan in *lcpA* mutant cells, representing features that are consistent with deficient attachment of rhamnan to peptidoglycan. Thus, our results strongly suggest that LcpA is the major LCP enzyme that catalyzes the covalent linkage of rhamnan to peptidoglycan, suggesting that under the circumstances examined, LcpA and LcpB play nonredundant roles. Interestingly, it was shown recently that the three *S. aureus* LCP proteins are able to attach WTA to peptidoglycan when tested *in vitro* in a reconstituted system with purified components, yet *in vivo*, the three proteins appear to have nonredundant functions where just one of them (LcpA) plays a major role (10).

When CWPS synthesis is impaired, as is the case for conditional *tagO* and *rgpA* mutants, substantial alterations of bacterial shape and impairment of cell division were observed. Also, although PSP-negative mutants are viable, they exhibit morphological defects such as long-chain formation and alteration of cell shape (11). These observations point out that CWPS play a crucial role in cell division and morphogenesis in *L. lactis* cells. Alterations in cell wall biogenesis that are associated with a reduced level or absence of CWPS have also been reported in streptococci (2, 23). Therefore, our results suggest the existence of mechanisms coordinating CWPS, peptidoglycan biosynthesis, and cell division.

## MATERIALS AND METHODS

### Bacterial strains, plasmids, and growth conditions.

The bacterial strains and plasmids used in this study are listed in Table S1. *L. lactis* strains were grown in M17 broth (Oxoid or Difco) supplemented with 5 g.liter^−1^ glucose (M17G) at 30°C. Where required, erythromycin, tetracycline, or chloramphenicol was added at a concentration of 5 µg/ml. *L. lactis* conditional mutants were precultured in the presence of 0.01 ng.ml^−1^ nisin (Sigma). To grow these mutants in the absence of nisin, bacteria from the preculture were harvested by centrifugation, washed once with 1 volume of sterile phosphate-buffered saline, and resuspended in 1 volume of M17 medium and this suspension was used to inoculate fresh M17G medium at 0.5% (vol/vol). To induce the expression of the genes placed under the control of the nisin-inducible promoter, nisin was added at 0.1 ng.ml^−1^ to the culture medium.

### Preparation of rhamnan from *L. lactis* MG1363 and 3107.

Cells were collected by centrifugation, washed twice with deionized water, resuspended in 5% TCA, and stirred for 48 h at 4°C; this was followed by centrifugation at 12,000 × *g*. The supernatant contained the PSP as reported previously (11). Rhamnan was extracted from the pellet containing cell debris essentially as described previously (30), with some modifications. The pellet was suspended in 0.01 N HCl, extracted at 100°C with stirring for 20 min, cooled, and centrifuged at 12,000 × *g*. The pellet was resuspended in 0.1 N HCl and treated as described above for another 20 min. The HCl extracts were deproteinated by addition of TCA (5%), dialyzed, and lyophilized to generate crude HCl extracts. The crude HCl extract (10 mg) was treated with 48% HF (100 µl) for 24 h at 4°C. After evaporation of HF under a stream of nitrogen, the residue taken in distilled H_2_O (1 ml) was fractionated on a Sephadex G-50 column (1 by 40 cm) and eluted with 0.1% acetic acid. Fractions (2 ml) were collected and assayed for neutral (31) and amino (32) sugars. Two peaks were detected, a broad peak (rhamnan) with a distribution coefficient (*K*_av_) of ∼0.2 to 0.5 positive only for neutral sugars and a second peak (fragments of PSP) with a *K*_av_ of ∼0.6 to 0.8 positive for both neutral and amino sugars. The distribution coefficient was calculated as *K*_av_ = (*V*_e_ − *V*0)/(*V*_t_ − *V*0), where *V*_e_ is the elution volume of the sample, *V*0 is the void volume, and *V*_t_ is the total volume of the column.

### Extraction of CWPS by HF and analysis by SEC-HPLC.

Bacteria were harvested from an exponentially growing culture at an optical density of 0.3, and cell walls were prepared as described previously (33). Briefly, heat-killed bacteria were boiled in 5% SDS and the pellet recovered by centrifugation was treated successively with pronase and trypsin to remove proteins and with RNase and DNase to remove nucleic acids. The resulting material, corresponding to purified cell walls (containing peptidoglycan and CWPS), was treated with 48% HF for 48 h at 4°C to extract CWPS. The samples were centrifuged at 20,000 × *g*, and the supernatant containing CWPS was dried under a stream of nitrogen. The residue was solubilized in Milli-Q H_2_O and lyophilized. Rhamnan and PSP oligosaccharides present in the sample were separated by SEC-HPLC with two columns in tandem (Shodex Sugar KS-804 and KS-803 columns; Showa Denko, Japan). Elution was performed with Milli-Q H_2_O, and detection of eluted compounds was performed with a refractometer (2414 Refractive Index Detector; Waters) and/or a UV detector at 206 nm. Fractions were collected and dried under vacuum. Their monosaccharide composition was determined by HPAEC-PAD, and they were further analyzed by MALDI-TOF MS.

### Digestion of cell walls by mutanolysin and analysis by SEC-HPLC.

Purified cell walls (6 mg) prepared as described above were digested in 750 µl of 25 mM sodium phosphate buffer (pH 5.5) with mutanolysin (2,500 U/ml; Sigma) for 24 h at 37°C while shaking on an Eppendorf Thermomix. Mutanolysin was inactivated by boiling for 3 min. The mixture was centrifuged at 20,000 × *g* for 15 min. The supernatant was recovered, and the constituents were separated by SEC-HPLC as described above. Fractions were collected, and fractions from peak I were pooled and lyophilized. Half of the peak I material was further treated with HF and subsequently analyzed by SEC-HPLC under conditions similar to those described above. The other half of the peak I material was analyzed by NMR spectroscopy.

### MALDI-TOF MS.

Purified fractions obtained by gel filtration on a Sephadex G-50 column or by SEC-HPLC were analyzed by MALDI-TOF MS with a Voyager-DE STR mass spectrometer (Applied Biosystems) with a 2,5-dihydroxybenzoic acid matrix.

### Monosaccharide analysis.

Monosaccharides were identified as reduced and acetylated derivatives (alditol acetates) by gas chromatography coupled to mass spectrometry (GC-MS) as described previously (34). The absolute configuration of the monosaccharides was determined by GC-MS analysis of acetylated (*R*)-2-butylglycoside derivatives as described previously (34). The products were analyzed by GC-MS and identified with standards prepared from monosaccharides with known configurations with (*R*)- and (*S*)-2-butanol. GC-MS was performed with a Trace GC ULTRA system (Thermo Scientific) equipped with an NMTR-5MS capillary column (30 m by 0.25 mm) by using a temperature gradient of 170°C (3 min) to 250°C at 5°C/min with a DSQ II MS detector.

The amount of CWPS extracted by HF from cell walls was determined by quantification of monosaccharides after acid hydrolysis with 4 M trifluoroacetic acid (TFA) for 3 h at 110°C. Monosaccharides were separated on a CarboPac PA20 column (Dionex) by HPAEC-PAD (ICS 5000 system; Thermo Fisher Scientific). Quantification was performed by comparison with standard amounts of each monosaccharide.

### Methylation analysis.

Methylation was performed with methyl iodide by the Ciucanu-Kerek procedure (35), as modified by Read et al. (36). The product was hydrolyzed with 4 M TFA (110°C, 3 h), dried, reduced with NaBD_4_, converted into alditol acetates, and analyzed by GC-MS as described above.

### NMR spectroscopy analysis.

NMR experiments were carried out with a Varian INOVA 500-MHz (^1^H) spectrometer with a 3-mm gradient probe at 25°C with an acetone internal reference (2.225 ppm for ^1^H and 31.45 ppm for ^13^C) by using standard pulse sequences for double-quantum COSY, total correlated spectroscopy (mixing time, 120 ms), rotating-frame nuclear Overhauser effect spectroscopy (mixing time, 500 ms), HSQC, and HMBC (100-ms long-range transfer delay). The acquisition time was kept at 0.8 to 1 s for H-H correlations and 0.25 s for HSQC, and 256 increments were acquired for t1. Assignment of spectra was performed with the Topspin 2 (Bruker Biospin) program for spectrum visualization and overlap.

HR-MAS NMR experiments were performed with an 18.8-T Bruker Avance III spectrometer. Experimental data were acquired with a ^1^H/^13^C/^31^P/^2^H probe with uniaxial gradients. Before analysis, cell pellets were washed twice with deuterium oxide (Euriso-Top, Gif-sur-Yvette, France). The 4-mm ZrO_2_ rotors (CortecNet, Paris, France) were filled with 50 μl of cell pellets, including 0.5 μl of acetone as an internal standard, and the final centrifugation rate was at 3,000 rpm. All spectra were recorded at 300 K, and the rotor spinning rate was 8 kHz. All experiments were sourced from the Bruker library pulse program, and delays and powers are optimized for each. For ^1^H-^13^C HSQC, the spectral widths were 12,820 Hz (^1^H) with 1,024 points for flame ionization detector resolution and 29,994 Hz (^13^C) for 400 scans, giving 12.5 and 75.0 Hz/point, respectively.

### TEM.

Pellets of bacteria were fixed with 2% glutaraldehyde in 0.1 M Na cacodylate buffer (pH 7.2) for 3 h at room temperature. After postfixation treatment with 1% osmium tetroxide containing 1.5% potassium cyanoferrate, pellets were dehydrated in solutions of increasing ethanol concentrations and embedded in Epon. Ultrathin sections were collected on 200-mesh copper grids and counterstained with lead citrate. Grids were examined with a Hitachi HT7700 electron microscope operated at 80 kV (Elexience, France), and images were acquired with a charge-coupled device camera (AMT).

### General molecular biology techniques.

Restriction enzymes (New England Biolabs), *Taq* DNA polymerase (MP Biomedicals), and Phusion High-Fidelity DNA polymerase (Thermo Fisher Scientific) were used as recommended by the manufacturers. Oligonucleotides were purchased from Eurogentec and are listed in Table S2. PCRs were performed with a Mastercycler PCR system (Eppendorf). DNA sequences were determined by GATC Biotech. Electrotransformation of *L. lactis* was performed as described previously (37).

10.1128/mBio.01303-17.2 TABLE S2  Primers used for gene inactivation. Download TABLE S2, DOCX file, 0.01 MB. Copyright © 2017 Sadovskaya et al. 2017 Sadovskaya et al. This content is distributed under the terms of the Creative Commons Attribution 4.0 International license.

### Construction of a conditional *tagO* mutant.

The chromosomal gene *tagO* (*llmg_1976/llnz_10205*) was placed under the control of the nisin-inducible P_*nisA*_ promoter (38). For this, a 1,342-bp fragment containing the complete *llmg_1976* gene starting at the ATG codon was amplified by PCR with Phusion High-Fidelity DNA polymerase with primers U-tagO_NcoI and l-tagO_XbaI employing MG1363 DNA as the template. The resulting amplicon was cloned downstream of the P_*nisA*_ promoter in the expression vector pNZ8048 (18). The resulting recombinant plasmid was used as the template to PCR amplify a 795-bp fragment encompassing the P_*nisA*_ promoter, a ribosome binding site, and the first 591 bp of *llnz_10205* with primers U-PnisA_HindIII and l-tagO-591_XbaI. The resulting fragment was digested with HindIII and XbaI and cloned into a HindIII/XbaI-linearized pJIM2374 vector (39). To lower the possible transcription of *llnz_10205* from the upstream erythromycin gene promoter, two strong terminators were cloned upstream of P_*nisA*_. For this, the plasmid was PCR amplified (inverse PCR) with primers Inv-pJIM2374_NotI and Inv-pJIM2274_XhoI, digested, and then ligated with a 243-bp NotI/XhoI-digested PCR fragment containing the T1-T2 terminator cluster of the *E. coli* rRNA operon (40). The resulting plasmid, pJIM_*tagO*, was produced in *E. coli* TG1 and used to transform *L. lactis* NZ9000 (18). Erythromycin-resistant clones were selected in the presence of 0.1 ng.ml^−1^ nisin, and plasmid integration was verified by PCR and sequencing. The selected *tagO* conditional mutant strain, which was named TIL1116, contains an intact copy of *tagO* in its chromosome under the transcriptional control of the nisin-inducible promoter.

### Construction of conditional *rgpA* and *lcpA* mutants.

For inactivation of *rgpA* (*llmg_0211/llnz_01100*) in *L. lactis* NZ9000, the two PCR products (a 307-bp P_*nisA*_ promoter fragment amplified with primers Pnis_1_PstI and Pnis_2 and a 371-bp *rgpA* gene fragment amplified with rgpA_3 and rgpA_4_SalI) were amplified from plasmid pVES5540 (41) and MG1363 genomic DNA, respectively, with Phusion High-Fidelity DNA polymerase. The PCR products were fused by the strand overlap extension method (42). After purification, the final PCR product was cut with PstI and SalI and cloned into plasmid pRV300 (43). The resulting plasmid, named pRV_Pnis_*rgpA*, contains a DNA fragment consisting of the P_*nisA*_ promoter, followed by part of the *rgpA* gene, encoding the first 123 amino acid residues. Plasmid pRV_Pnis_*rgpA* was introduced into NZ9000 by electroporation. Strain VES6945, containing the *rgpA* gene under the transcriptional control of the nisin-inducible promoter, was selected among erythromycin-resistant clones and verified by PCR and nucleotide sequence determination for a single-crossover event.

For inactivation of *lcpA* (*llmg_0461/llnz_02385*) in *L. lactis* NZ9000, two PCR products (a 307-bp fragment amplified with Pnis_1_PstI and Pnis_2 and a 391-bp fragment amplified with Llmg_0461_3 and Llmg_0461_4_SalI) were amplified from plasmid pVES5540 (41) and MG1363 genomic DNA, respectively, with Phusion High-Fidelity DNA polymerase. The PCR products were fused by the strand overlap extension method (42). After purification, the final PCR product was cut with PstI and SalI and cloned into plasmid pRV300 (43). The resulting plasmid, pRV_Pnis_*lcpA*, contains a DNA fragment of 655 bp corresponding to 295 bp of nisin-inducible promoter DNA, followed by the 360 bp encoding the first 120 residues of LcpA. Plasmid pRV_Pnis_*lcpA* was electroporated into NZ9000. Strain VES6320, containing the *lcpA* gene under the transcriptional control of the nisin-inducible promoter, was selected among erythromycin-resistant clones and verified by PCR and nucleotide sequence determination for a single-crossover event.

### Construction of an *lcpB* deletion mutant.

An internal deletion of the *lcpB* (*llmg_0733/llnz_03815*) gene was constructed with the pORI280 (lacZ+)/pVE6007 two-plasmid system (44, 45). For this purpose, fragments flanking the deletion site in the *lcpB* gene were PCR amplified from *L. lactis* MG1363 genomic DNA with primer pairs 733BamHI-733XmaIR and 733XmaIF-733XbaI. The PCR-amplified fragments were digested with XmaI and ligated. The resulting fragment was PCR amplified with primers 733BamHI and 733XbaI, digested with BamHI and XbaI, and ligated to BamHI-XbaI-digested pORI280. The ligation mixture was transformed into *L. lactis* LL302, an MG1363 derivative carrying a chromosomal copy of *repA*, required for pORI280 replication (46). Recombinant pORI280 carrying a fragment with a deletion in the *lcpB* gene was transformed into an NZ9000 derivative carrying pVE6007 (a thermosensitive plasmid encoding a chloramphenicol resistance determinant), and erythromycin (2.5 µg.ml^−1^)- and chloramphenicol (5 µg.ml^−1^)-resistant clones were selected. Integration of the pORI280 derivative into the chromosome of the selected strain was obtained after overnight growth in M17G liquid medium supplemented with erythromycin at 37°C, which is a nonpermissive temperature for pVE6007 replication. The culture was subsequently plated on M17G agar with erythromycin, and six independent chloramphenicol-sensitive clones were isolated and grown in M17G without antibiotics for at least 100 generations. Strain PAR152 carrying the deletion of *lcpB* was then selected as a white colony on M17G agar supplemented with 5-bromo-4-chloro-3-indolyl-β-d-galactopyranoside (Euromedex). The presence of the *lcpB* deletion was verified by PCR and nucleotide sequence determination.

### Construction of a conditional *lcpA* mutant in the *lcpB* deletion mutant.

The double mutant was constructed by creating the conditional *lcpA* mutation (described above) in the PAR152 *lcpB* deletion mutant. To achieve this, plasmid pRV_Pnis_*lcpA* was electroporated into PAR152. Strain PAR136, containing the *lcpA* gene under the transcriptional control of the nisin-inducible promoter, was selected among erythromycin-resistant clones and verified by PCR and nucleotide sequence determination for a single-crossover event.
