# Supplementary material for: Another Brick in the Wall: a Rhamnan Polysaccharide Trapped inside Peptidoglycan of Lactococcus lactis
Source: mBio. 2017 Sep 12;8(5):e01303-17. doi: 10.1128/mBio.01303-17 (PMC5596347; doi:10.1128/mBio.01303-17)
Supplement: TABLE S1 [file mbo004173460st1.docx]

**TABLE S1**. *L. lactis* strains and plasmids used in this study

| Strains and plasmids | Relevant features | Source/  reference |
| --- | --- | --- |
| ***L. lactis*** |  |  |
| MG1363 | *L. lactis* subsp. *cremoris*, plasmid-free and prophage-cured derivative of NCDO712 | ([1](#_ENREF_1)) |
| NZ9000 | MG1363 derivative *pepN::nisRK,* allows nisin-inducible gene expression |  |
| 3107 | *L. lactis* subsp. *cremoris* | ([2](#_ENREF_2)) |
| LL302 | MG1363 derivative carrying a chromosomal copy of *repA* | ([3](#_ENREF_3)) |
| VES5748 | MG1363 spontaneous mutant, C to T transition in *llmg_0226* | ([4](#_ENREF_4)) |
| NZ9000-GT1 | NZ9000 with GAATTC insert in *LLNZ_01145* (corresponding to *llmg_0220*), resulting in an in-frame TGA stop codon | ([2](#_ENREF_2)) |
| NZ9000-GT1/pPTPiC2 | NZ9000-GT1 containing pPTPiC2 plasmid | ([2](#_ENREF_2)) |
| VES6945 | Ery^r^, NZ9000 *rgpA* conditional mutant, obtained by pRV_Pnis_*rgpA* single cross-over (SCO) integration | This study |
| TIL1116 | Ery^r^, NZ9000 *tagO* (*llnz_10205*) conditional mutant, obtained by pJIM*_tagO* SCO integration | This study |
| VES6320 | Ery^r^, NZ9000 *lcpA (llnz_02385)* conditional mutant, obtained by pRV_Pnis_*lcpA* SCO integration | This study |
| PAR152 | NZ9000 *ΔlcpB* (*llnz_03815*) | This study |
|  |  |  |
|  |  |  |
| PAR136 | Ery^r^, NZ9000 *ΔlcpB lcpA* conditional mutant, obtained by pRV_Pnis_*lcpA* SCO integration in NZ9000 *ΔlcpB* | This study |
| **Plasmids** |  |  |
| pNZ8048 | Cm^r^, shuttle vector containing P*_nisA_* with a downstream start codon | ([5](#_ENREF_5)) |
| pJIM2374 | Ery^r^, derivative of pORI28 non-replicative vector in *L. lactis*, carrying *luxAB* gene from *Vibrio harveyi* | ([6](#_ENREF_6)) |
| pRV300 | Em^R^ , Amp^r^, pBluescript derivative | ([7](#_ENREF_7)) |
| pORI280 | Em^R^, ori^+^, LacZ^+^, derivative of pWV01, replicates only in strains providing *repA* in trans | ([8](#_ENREF_8)) |
| pVE6007 | Cm^R^, thermosensitive derivative of pWV01 | ([9](#_ENREF_9)) |
| pPTPiC2 | Tet^r^, plasmid pPTPi carrying genes *3107_003, 3107_004 and 3107_005* from *L. lactis* 3107 under the control of P*nisA* promoter | ([2](#_ENREF_2)) |
| pJIM*_tagO* | Ery^r^, pJIM2374 derivative carrying a 591-bp fragment from *llnz_10205* under P*_nisA_* promoter control and two strong terminators upstream | This study |
| pRV_Pnis_*rgpA* | Ery^r^, pRV300 derivative carrying 371 bp of *llnz_01100* fragment under P*_nisA_* promoter control | This study |
| pRV_Pnis_*lcpA* | Ery^r^, pRV300 derivative carrying 360 bp of *llnz_02385*  fragment under P*_nisA_* promoter control | This study |
|  |  |  |
|  |  |  |

References:

1. **Gasson MJ.** 1983. Plasmid complements of *Streptococcus lactis* NCDO 712 and other lactic streptococci after protoplast-induced curing. J Bacteriol **154:**1-9.

2. **Ainsworth S, Sadovskaya I, Vinogradov E, Courtin P, Guerardel Y, Mahony J, Grard T, Cambillau C, Chapot-Chartier MP, van Sinderen D.** 2014. Differences in lactococcal cell wall polysaccharide structure are major determining factors in bacteriophage sensitivity. MBio **5:**e00880-00814.

3. **Leenhouts K, Bolhuis A, Venema G, Kok J.** 1998. Construction of a food-grade multiple-copy integration system for *Lactococcus lactis*. Appl Microbiol Biotechnol **49:**417-423.

4. **Chapot-Chartier MP, Vinogradov E, Sadovskaya I, Andre G, Mistou MY, Trieu-Cuot P, Furlan S, Bidnenko E, Courtin P, Pechoux C, Hols P, Dufrene YF, Kulakauskas S.** 2010. The cell surface of *Lactococcus lactis* is covered by a protective polysaccharide pellicle. J Biol Chem **285:**10464-10471.

5. **Kuipers OP, de Ruyter PGGA, Kleerebezem M, de Vos WM.** 1998. Quorum sensing-controlled gene expression in lactic acid bacteria. J Biotechnol **64:**15-21.

6. **Delorme C, Ehrlich SD, Renault P.** 1999. Regulation of expression of the *Lactococcus lactis* histidine operon. J Bacteriol **181:**2026-2037.

7. **Leloup L, Ehrlich SD, Zagorec M, Morel-Deville F.** 1997. Single-crossover integration in the *Lactobacillus sakei* chromosome and insertional inactivation of the *ptsI* and *lacL* genes. Appl Environ Microbiol **63:**2117-2123.

8. **Leenhouts K, Buist G, Bolhuis A, ten Berge A, Kiel J, Mierau I, Dabrowska M, Venema G, Kok J.** 1996. A general system for generating unlabelled gene replacements in bacterial chromosomes. Mol Gen Genet **253:**217-224.

9. **Law J, Buist G, Haandrikman A, Kok J, Venema G, Leenhouts K.** 1995. A system to generate chromosomal mutations in *Lactococcus lactis* which allows fast analysis of targeted genes. J Bacteriol **177:**7011-7018.
