# Supplementary material for: Another Brick in the Wall: a Rhamnan Polysaccharide Trapped inside Peptidoglycan of Lactococcus lactis
Source: mBio. 2017 Sep 12;8(5):e01303-17. doi: 10.1128/mBio.01303-17 (PMC5596347; doi:10.1128/mBio.01303-17)
Supplement: TABLE S2 [file mbo004173460st2.docx]

**TABLE S2.** Primers used for gene inactivation

| **Primer name** | **Sequence*^a^*** | | |
| --- | --- | --- | --- |
| **Primers used for *tagO* conditional mutation construction** | | |  |
| U-tagO-NcoI | 5’-CATGCCATGGTTGATACATTAAAAGAAATC-3’ | | |
| L-tagO_XbaI | 5’-CTAGTCTAGATTATTTTTTATGTCGACTTCTTCG-3’ | | |
| U-PnisA_HindIII | 5’-CCCAAGCTTAGATCTAGTCTTATAACTATAC-3’ | | |
| L-tagO-591_XbaI | 5’-CTAGTCTAGACAAGTCCGTCTAAACCATC-3’ | | |
| Inv-pJIM2374_NotI | 5’-ATAAGAATGCGGCCGCGACAGCTTCCAAGGAGC-3’ | | |
| Inv-pJIM2274_XhoI | 5’-CCGCTCGAGAGTAGTATACCTAATAATTTATC-3’ | | |
| **Primers used for *rgpA* conditional mutation construction** | | |  |
| Pnis_1_PstI | 5’-GATCCTGCAGTGAGATAATGCCGACTGTAC-3’ | | |
| Pnis_2 | 5’-CATCTCGAGTGCCTCCTTA-3’ | | |
| rgpA_3 | 5’-ATTATAAGGAGGCACTCGAGatgaaaaaacacgtttttatcatt-3’ | | |
| rgpA_4_SalI | 5’-GATCgtcgaccgccaagtttgtgaattttct-3’ | | |
| **Primers used for *lcpA* conditional mutation construction** | | |  |
| Pnis_1_PstI | 5’- GATCCTGCAGTGAGATAATGCCGACTGTAC -3’ | | |
| Pnis_2 | 5’- CATCTCGAGTGCCTCCTTA-3’ | | |
| Llmg_0461_3 | 5’-ATTATAAGGAGGCACTCGAGATGAAGCTTTGGATAAAAACAC-3’ | | |
| Llmg_0461_4_SalI | 5’-GATCGTCGACTAGAGACGATGTTTCCGTCA-3’ | | |
| **Primers used for *lcpB* deletion** | |  |  |
| 733BamHI | 5’-tgttgtggatccaaatggtcaagaagtgctgg-3’ | | |
| 733XmaIR | 5’-tgttgtcccgggataattcctgcaaaaacttgc-3’ | | |
| 733XmaIF | 5’-tgttgtcccgggaaagctcaagaactccttgg-3’ | | |
| 733XbaI | 5’-tgttgttctagatcgttaacttccttatcttcc-3’ | | |

*^a^* Restriction sites introduced in the primers are underlined
